# Tobacco dependence affects determinants related to quitting intention and behaviour

**DOI:** 10.1038/s41598-021-99766-z

**Published:** 2021-10-12

**Authors:** Haoxiang Lin, Meijun Chen, Qingping Yun, Lanchao Zhang, Chun Chang

**Affiliations:** Department of Social Medicine and Health Education, School of Public Health, Peking University Health Science Center, 38 Xueyuan Rd, Haidian District, Beijing, China

**Keywords:** Public health, Health policy

## Abstract

This study uses protection motivation theory (PMT) to examine the quitting intentions and behaviours of smokers who have varying levels of nicotine dependence. Our goals are to identify the psychological factors that influence smoking cessation and to provide valuable evidence to promote theory-guided interventions. This is a cross-sectional study that was conducted from July to August 2020. Participants were randomly selected on the streets of 26 provinces on mainland China. Data were collected via face-to-face interviews. Our analysis was conducted in three steps. First, we employed descriptive statistics to present the overall characteristics of our sample. Second, we analysed the association between PMT constructs and quitting intentions stratified by nicotine dependence. Third, we tested how quitting intentions were associated with quitting behaviours in each subgroup using logistic regression models. For intention, almost all the PMT constructs were significantly associated with quitting intention in the low-dependence group. For the moderate- and high-dependence groups, only perceived vulnerability (coefficient = 0.35, P = 0.04) was positively associated with quitting intention. For behaviour, we found a stronger association between quitting intention and behaviour in the low-dependence group (Coef. = 1.67, P = 0.00) than for the other groups. We found a significant association between e-cigarette use and quitting behaviour only in the low-dependence group (Coef. = 1.34, P = 0.00). Coefficients for the moderate- and high-dependence groups were not statistically significant. Smokers at various levels of nicotine dependence have different psychological factors that influence their intentions to stop smoking. Quitting intention was more significantly associated with quitting behaviour for the low nicotine-dependence group than for the other groups. More convincing research is necessary to determine how e-cigarette use affects quitting behaviour in the long term.

## Introduction

In recent years, China has taken some important steps toward reducing tobacco use. However, studies have found that efforts have met with limited success in the past decade. According to the China Adult Tobacco Survey (CATS), the smoking prevalence for males was 52.9% in 2010, 52.1% in 2015 and 50.5% in 2018, while the prevalence for females was 2.4% in 2010, 2.7% in 2015 and 2.1% in 2018^1–3^. One of the reasons for this continued high prevalence of smoking is the low quitting intentions of current smokers (only 5.6% smokers report wanting to stop smoking within a month)^3^. Quitting intentions are a strong predictor of attempts to quit, and attempts to quit are a strong predictor of successful smoking cessation; this, in turn, works in conjunction with quitting intention to contribute to long-term abstinence^4^. Therefore, it is critically meaningful to understand the factors that are associated with quitting intention and behaviours to help smokers stop smoking. In this study, quitting intention refers to the desire to stop smoking. Quitting behaviours refer to the actual attempt to stop smoking. We provide more specific information on how to measure quitting intention and behaviour in the Methods section.

A large number of studies have shown that many factors can potentially influence the intention to stop smoking, but most previous studies focused only on socioeconomic and demographic factors^5–8^. Psychological factors, such as threat appraisal and coping appraisal pathways of smoking behaviour that might suggest interventions, remain understudied. In addition, when assessing quitting behaviour-related factors, few studies are grounded in or guided by specific health behaviour theory, such as the health belief model, the theory of planned behaviour, the elaboration likelihood model, and social cognitive theory. As a result, the issue of how to effectively integrate the identified factors into theory-based smoking cessation strategies is almost untouched by researchers.

Many past studies have taken tobacco dependence and other psychological measures as same-level factors^9,10^. However, an increasing number of studies have found that nicotine dependence is the most consistent and significant variable associated with quitting attempts and that it can also influence other psychological factors^5,11,12^. Thus, it is important to conduct further research based on different tobacco dependence statuses.

### Evidence prior to this study

In the previous year, psychological factors related to quitting activities have received some attention. Li et al. used International Tobacco Control (ITC) China Survey data and found that higher quitting self-efficacy and more immediate intentions to quit are independent predictors of quitting attempts^9^. A clinical study found that those who have fewer past failed attempts to quit and believe that it is not too late to quit are more likely to consider quitting^10^. Other researchers have found that higher perceptions of severity and vulnerability to smoking-related diseases are associated with higher odds of quitting attempts^13^. A cohort study from six European countries found that a lower level of perceived addiction and health concerns and setting a good example for children are among the most important predictors of smoking cessation^14^.

With regard to nicotine dependence, the association with quitting intentions and quitting attempts is well established. On the one hand, studies have found that high nicotine dependence is associated with failure to quit attempts^12,15^. Attempts to quit are significantly associated with low nicotine dependence (AOR = 5.85, CI 2.85–12.00)^16^. On the other hand, nicotine dependence is negatively related to intentions to quit^17^. Tobacco users with a high Fagerstrom test for nicotine dependence (FTND) score are 1.83 and 3.30 times less likely to intend to quit^18^. In addition, other researchers have found that nicotine dependence not only has a direct impact on quitting attempts but also has a joint influence on quitting intention to moderate the effect of quitting attempts among Dutch students^19^. Therefore, we believe that nicotine dependence may affect the relationship between quitting intention and quitting attempt among Chinese adults.

### Theoretical framework

**Figure 1:**
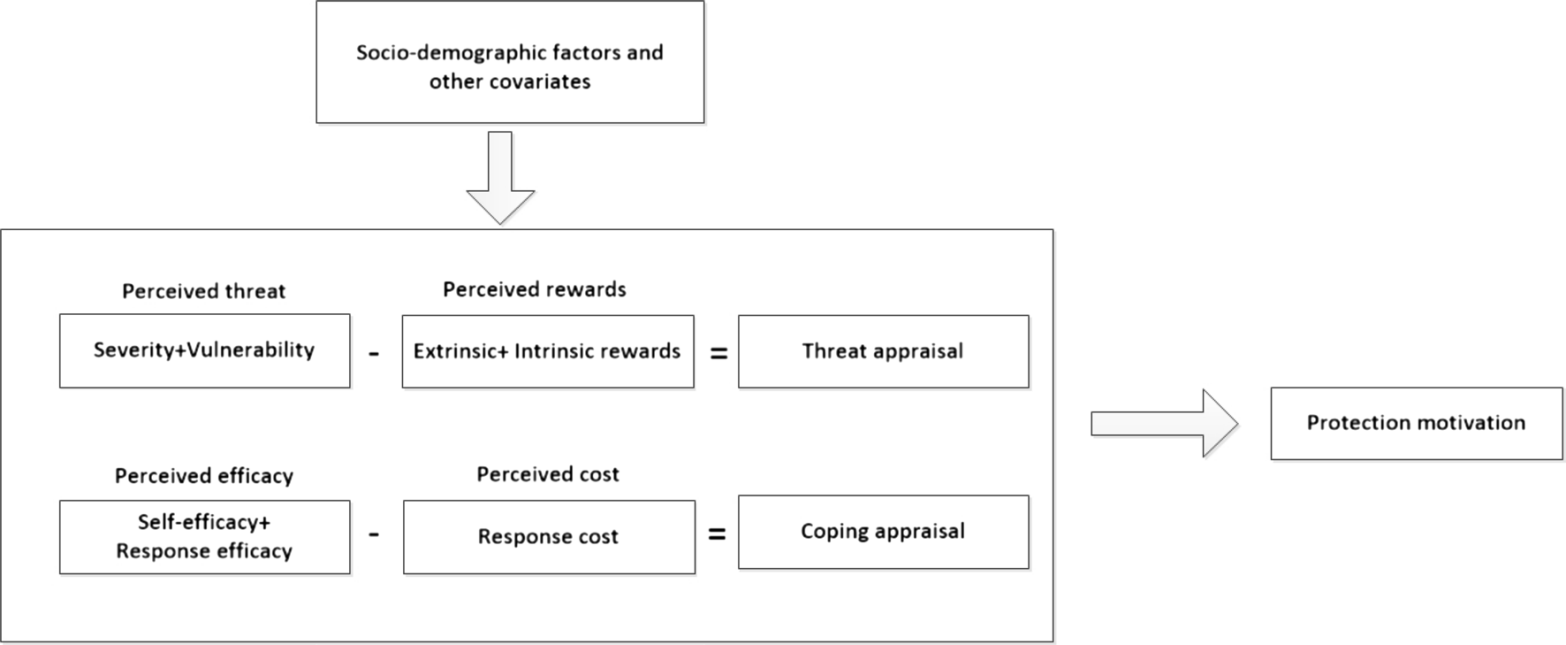
Protection motivation theory framework.

Protection motivation theory (PMT) is a famous theory of behaviour change^20^. The basic idea behind this theory is that prevention behaviour is driven by an evaluation of the risk and coping response^21^. As shown in Fig. 1, PMT has seven elements: perceived severity, perceived susceptibility, intrinsic and extrinsic rewards, response efficacy, self-efficacy and response costs. Threat appraisal is one dimension and serves as an evaluation of maladaptive behaviours. Stronger motivation for a specific health behaviour (such as quitting intention) can be expected if the perceived severity and vulnerability are high and the rewards are low.

Coping appraisal is also a potential pathway for ‘protection motivation’ and serves as an evaluation of a person’s ability to manage and avoid the threat. Response efficacy and self-efficacy are expected to enhance protection motivation, whereas response costs are expected to reduce motivation^22–24^.

Among the major theories currently used in the field of addiction, PMT may be particularly well suited for understanding smoking behaviour. It has been widely used in the West as a framework for researchers to investigate smoking behaviour because its threat and coping appraisal pathways are particularly useful in explaining why people engage in risky behaviour^25^. The balance between such pathways involves comparing not only perceived threats of smoking but also perceived rewards of smoking and perceived response costs of smoking cessation. Thus, it is particularly useful to explain why people decide to become smokers, despite the well-known health risks of smoking. Some studies have been conducted among adolescents based on constructs of PMT, such as that by Johannes et al., who found that only one part of PMT (self-efficacy) has predictive validity regarding adolescents’ smoking intentions and related behaviours^26^. Karen et al. found that two of seven PMT factors are significantly correlated with smoking intention among adolescents^27^.

As mentioned, assessing quitting behaviour by health theory can provide a more comprehensive evaluation framework of influential psychological variables and thus provide valuable evidence for further smoking cessation interventions. One meta-analysis, which analysed six studies based on PMT and smoking cessation, concluded that coping appraisal variables show stronger effects regarding the prevention of smoking and smoking cessation than threat appraisal variables^28^. Other researchers have found that perceived rewards of tobacco use, especially intrinsic rewards, are consistently positively related to smoking intentions and behaviour^27–29^.

## Hypotheses

In this study, we use PMT to examine the psychological-level determinants of quitting intention and behaviour related to varying levels of nicotine dependence, with the goal of providing valuable evidence to promote theory-guided cessation interventions. First, we want to assess how well the different constructs of this theory are associated with quitting intention and how this relationship varies across smokers who have different nicotine dependence levels. Then, we examine how quitting intention and other identified factors are associated with actual quitting behaviour.

Based on the available literature, we hypothesize the following. (1) Smokers with different nicotine dependence levels have different psychological determinants of quitting intention. (2) Among participants who have various levels of nicotine dependence, there will be a difference in the relationship between quitting intention and behaviour.

This paper extends prior research along two dimensions. First, to the best of our knowledge, this is the first study to evaluate the association between quitting intention and its psychological determinants, especially in relation to nicotine dependence, in the context of behavioural change theory. Second, many studies have left the relation between psychological determinants and actual behaviour untested because it is often difficult or even impossible to measure effects on behaviour. We recognize that intentions are important, but measuring the intention alone provides only a partial picture. Thus, we test psychological determinants and the target behaviour simultaneously.

## Methods

### Study population and procedure

This is a cross-sectional study that was conducted from July to August 2020. The School of Public Health at Peking University Health Science Centre sent investigators to 26 provinces on mainland China. Participants were randomly selected on the street in large business districts and residential areas in urban regions. Data were collected via face-to-face interviews. Participants took approximately 10 min to complete all the assessments. We did not provide any incentives for participation. Adult daily smokers who had smoked for more than one year and had lived in the city ≥ 5 years were considered eligible for participation. The questionnaires were designed by Peking University and consisted of 50 items assessing demographic and sociological information, smoking and quitting information, individual health literacy and lifestyle information and aspects related to PMT.

### Ethical considerations

The study protocol was approved by the ethics commission of Peking University Health Science Centre (Ethical approval number: IRB00001052-18055). All methods were performed in accordance with the ethical guidelines and regulations. Informed consent was obtained. All participants were informed that the statistical analyses would be conducted anonymously and that their information would be used for research purposes and published.

We state that there are no conflicts of interest.

### Measurements

#### The measurement of quitting intentions and behaviour

Quitting intentions were measured by the transtheoretical model (TTM). In the survey, participants were asked, “Are you going to quit smoking? A: yes, within a month, B: yes, within 6 months, C: yes, but not within 6 months, D: no plan for quitting.” The participants who chose A or B were classified as having quitting intentions. The reason is that according to the TTM, if a smoker does not want to quit within 6 months, he or she is classified as being in the precontemplation stage^30^. Quitting behaviour was measured by the following question. The respondents were asked, ‘Have you tried to quit smoking this year? A: Yes, B: No’.

### The measurement of nicotine dependence

The Fagerstrom Test for Nicotine Dependence is a 6-item scale that measures physical dependence on nicotine^31^. In the survey, the participants were asked ‘(1) How soon after you wake up do you smoke your first cigarette? (2) Do you find it difficult to refrain from smoking in places where it is forbidden? (3) Which cigarette would you hate most to give up? (4) How many cigarettes per day do you smoke? (5) Do you smoke more frequently during the first hours after waking than during the rest of the day? (6) Do you smoke if you are so ill that you are in bed most of the day?’ Each answer was coded from 0 to 3. Scores on the test ranged from 0 to 10 (0–3 indicated low nicotine dependence; 4–6 indicated moderate dependence; and ≥ 7 indicated high dependence)^32^.

### The measurements of PMT constructs

PMT constructs were assessed using the PMT scale, which was based on the work of Xu et al.^30^. We also improved and adjusted some of the questions to make them fit for measures of quitting intention. Specifically, the scale comprised 21 items using a 7-point Likert-type scale with responses ranging from 1 (definitely disagree) to 7 (definitely agree). Each construct subscale includes three items, and we computed the mean as the subscale score. We have published the details of this scale and evaluation process elsewhere^4^.

Perceived severity was measured by the following: ‘The earlier a person starts smoking, the greater the harm’, ‘More smokers get sick than nonsmokers’, and ‘Smokers die earlier than nonsmokers’. Perceived vulnerability was measured by: ‘I would become addicted if I smoked’, ‘I would get sick if I smoked’, and ‘If I smoked, I may die earlier’. Intrinsic rewards were measured by: ‘Smoking makes people feel comfortable’, ‘Smoking helps people concentrate’, and ‘Smoking enhances brainwork’. Extrinsic rewards were measured by: ‘Smokers look cool and fashionable’, ‘Smoking is good for social networking’, and ‘The life of a smoker is happier than that of a nonsmoker’. Self-efficacy was measured by: ‘I am confident that I can quit smoking successfully’, ‘I have the ability to stop smoking’, and ‘I think stopping smoking is easy for me’. Response efficacy was measured by: ‘People will feel good if they do not smoke’, ‘People will be less likely to get sick if they do not smoke’, and ‘Quitting smoking is good for disease recovery’. Response cost was measured by: ‘A person may be isolated if he or she stops smoking’, ‘Refusing a cigarette offer is very impolite’, and ‘One will miss the enjoyment if he or she quits smoking’.

### Other variables

We also collected several variables of individual characteristics and sociodemographic information, including sex, age, marital status, ethnicity, education, yearly income, and chronic diseases. Chronic disease was assessed by the question: “Do you have medical institution-confirmed chronic diseases? Yes or No?” Life satisfaction was assessed by the question: “Are you satisfied with your current life? Yes or No?” Alcohol use was assessed by the question, “How often do you drink alcohol?” The response options were A: everyday, B: always C: sometimes, D: I never drink alcohol. These four groups were collapsed into two groups of nondrinkers (D) and drinkers (A or B or C) and jobs. Furthermore, we assessed smoking status, including the number of cigarettes per day and e-cigarette usage. E-cigarette usage was assessed by the question: “Do you currently use e-cigarettes? Yes or No”, and smoking cessation information (i.e., quitting attempts and quitting methods).

### Data analysis

Our data analysis was conducted in three steps. First, we employed descriptive statistics to present the overall characteristics of our sample. Second, we analysed the association between PMT constructs and quitting intention stratified by nicotine dependence. Third, we examined how quitting intention was associated with quitting behaviour in each subgroup.

*Step 1*: Categorical variables were presented as counts and percentages. Comparisons of PMT construct scores between different groups were performed.

*Step 2*: Binary logistic regression models were used to test the association between seven PMT constructs and quitting intention. All the samples were classified as low nicotine dependence, moderate dependence and high dependence.

*Step 3*: To examine intention and behaviour simultaneously, we used binary logistic regression models to examine how intention was associated with actual behaviour in each nicotine dependence group. The dependent variable was quitting behaviour. The explanatory variables were quitting intention, e-cigarette use and other control variables. We also stratified our sample by nicotine dependence. Univariate and multivariate analyses were performed.

We chose appropriate demographic covariates by correlation > 0.30 with both independent and outcome variables. We controlled for sex, education attainment, and alcohol consumption in all the models.

These analyses were performed with SPSS V.11.0 (SPSS, Chicago, Illinois, USA).

### Ethics approval and consent to participate

This study was approved by the Peking University. Informed consent has been obtained.

## Results

### Descriptive statistics

We approached 738 smokers. After screening, 613 were identified as eligible for interviews. The participants were from 26 provinces on mainland China. Table 1 shows the descriptive statistics for the overall sample. The mean age of the study population was 37.95 ± 14.31 (mean values and SD). Males accounted for 91.7 percent of the sample (n = 562). A total of 297 (48.5%) study subjects wanted to quit tobacco. The majority of the people had low nicotine dependence (70.0%).

**Table 1:** Descriptive statistics for the overall sample.

Demographics	n/%
**Age**	
18–29	240 (39.2)
30–39	86 (14.0)
40–49	133 (21.7)
50 and above	154 (25.1)
Mean (SD)	37.95 (14.31)
**Sex**	
Male	562 (91.7)
Female	51 (8.3)
**Ethnicity**	
Han	544 (88.7)
Other	69 (11.3)
**Marriage**	
Single	230 (37.5)
Married	359 (58.6)
Divorced or widowed	24 (3.9)
**Educational attainment**	
Master/above	41 (6.7)
Bachelor	319 (52.0)
High school	129 (21.0)
Middle school	79 (12.9)
Primary school/lower	45 (7.3)
**Number of cigarettes per week**	
1–50	287 (46.8)
51–100	125 (20.4)
101–150	163 (26.6)
> 150	38 (6.2)
Mean (SD)	74.86 (90.48)
**Have quitting intention**	
Yes	297 (48.5)
No	316 (51.5)
**Quitting behaviour**	
Yes	112 (18.3)
No	501 (81.7)
**Nicotine dependence**	
Low	429 (70.0)
Moderate	148 (24.1)
High	36 (5.9)
**Have chronic disease**	
Yes	104 (17.0)
No	509 (83.0)
Total	613

Table 2 shows the subscale score of PMT. Moderate and high-dependence smokers had lower mean scores (SD) of perceived severity of smoking-related diseases, self-efficacy and response efficacy of quitting and higher mean scores (SDs) of intrinsic and extrinsic rewards of smoking and response costs of quitting.

**Table 2:** Item score of the PMT.

Item and primary subconstructs	Mean (SD)	
	Low dependence	Moderate and high dependence
Perceived severity	5.59 (1.44)	5.23 (1.74 )
Perceived vulnerability	4.74 (1.49)	5.11 (1.49)
Intrinsic rewards	4.81 (1.56)	5.44 (1.43)
Extrinsic rewards	3.54 (1.37)	3.57 (1.48)
Self-efficacy	4.42 (1.82)	3.36 (1.84)
Response efficacy	4.96 (1.30)	4.64 (1.44)
Response cost	3.41 (1.57)	3.84 (1.63)

### The association between seven PMT constructs and quitting intention

The association between the seven PMT constructs and quitting intention was different depending on nicotine dependence level (Table 3). For the low-dependence group, almost all the PMT constructs were significantly associated with quitting intention. Stronger intentions were significantly associated with higher perceived severity (coefficient = 0.27, P = 0.00), vulnerability (coefficient = 0.19, P = 0.04), self-efficacy (coefficient = 0.17, P = 0.01), and response efficacy (coefficient = 0.22, P = 0.03) but were inversely associated with intrinsic rewards (coefficient = -0.19, P = 0.02).

**Table 3:** Logistic regression models of the quitting intention regressed on the PMT variables by tobacco dependence.

	Quitting intention			
	Low dependence		Moderate and high dependence	
	Coefficient	P value	Coefficient	P value
Severity	0.27*	0.00	− 0.17	0.84
Vulnerability	0.19*	0.04	0.35*	0.04
Intrinsic rewards	− 0.19*	0.02	− 0.27	0.05
Extrinsic rewards	0.02	0.84	0.05	0.76
Self-efficacy	0.17*	0.01	0.04	0.72
Response efficacy	0.22*	0.03	0.19	0.21
Response cost	− 0.13	0.14	− 0.25	0.07
R^2^	0.23		0.24	

We controlled for sex, education attainment, alcohol drinking in all the models.

*p < 0.05.

For the moderate- and high-dependence groups, only perceived vulnerability (coefficient = 0.35, P = 0.04) was positively associated with quitting intention.

To examine the role of tobacco dependence directly, we replaced the dependent variable (PMT variables) with FTND scores and other control variables. The coefficient of FTND scores was -0.08 (P = 0.03), which suggests that quitting intention is negatively associated with tobacco dependence.

### The role of quitting intention to behaviour

To evaluate the effect of quitting intention on behaviour, we changed the dependent variable to quitting behaviour and accounted for tobacco dependence, e-cigarette use and demographic factors. The results are shown in Table 4. It is worth noting that we found a stronger association between quitting intention and behaviour in the low-dependence group (Coef. = 1.67, P = 0.00) than in the other groups. A similar pattern can be observed for the association between e-cigarette use and quitting behaviour. We only found a significant association between e-cigarette use and quitting behaviour in the low-dependence group (Coef. = 1.34, P = 0.00). The coefficients of the moderate- and high-dependence groups were not statistically significant.

**Table 4:** Logistic regression models of the quitting behaviour regressed on quitting intention and e-cigarette use by tobacco dependence.

	Quitting behaviour			
	Low dependence (coefficient and P value)		Moderate and high dependence (coefficient and P value)	
	Univariate	Multivariate	Univariate	Multivariate
Quitting intention	1.61*(0.00)	1.67*(0.00)	1.51*(0.00)	1.47*(0.00)
E-cigarette use	1.10*(0.00)	1.34*(0.00)	0.56(0.16)	0.54(0.21)
R^2^		0.21		0.19

We controlled for sex, education attainment, alcohol drinking in all the models.

*p < 0.05.

The estimated R^2^ indicated that these models accounted for 19–24% of the variance in quitting intentions or behaviours. In addition, with all the model fit tests being significant, we believe the overall picture is meaningful.

## Discussion

This study joins the debate in recent years on the determinates related to smoking cessation and provides evidence from a more general perspective. These Chinese data provide a comprehensive picture of how PMT variables are associated with quitting intention and how intention affects quitting behaviour for smokers with different levels of nicotine dependencies. The study provides new and potentially important information. It successfully shows that the relationship between PMT variables and quitting intention is substantially impacted by nicotine dependence level. In other words, smokers with varying degrees of nicotine dependence have different psychological determinants of quitting intention (first hypothesis).

The results of the present study showed that most PMT variables were significantly associated with protection motivation for low nicotine-dependent smokers. However, consistent with other studies, not all PMT measures had the same strength in predicting quitting intention^28,33^. A previous meta-analysis showed that coping appraisal variables, especially self-efficacy, are the strongest predictors of protection motivation and behaviour^26^. Notably, almost none of these factors can promote smoking intention for high-dependence smokers. These findings highlight the necessity of accounting for smokers’ nicotine dependence levels when designing smoking cessation interventions. The findings could also encourage policymakers and health practitioners to implement tailored behavioural interventions and thus contribute to achieving better smoking-reduction outcomes.

It is worth noting that actual quitting behaviour is affected by quitting intention, tobacco dependence and e-cigarette usage. However, quitting intention is more significantly associated with quitting behaviour only for the low dependence group (second hypothesis). This finding is consistent with previous research showing that greater nicotine dependence is associated with weaker motivation to quit smoking and lower abstinence rates. While we recognize that intentions are important, as they ‘get the ball rolling’^31^, these findings indicate that the association between quitting intention and behaviour is influenced by nicotine dependence level. Given that many highly nicotine-dependent smokers may have difficulty pursuing abstinence depending on motivation intervention alone, our study calls for the promotion of accessible and affordable intensive smoking cessation interventions or smoking cessation medications for this population. Our previous study found that the limited use of smoking cessation medications in China is a problem. The main barrier to accessing these medications is their cost. An obvious remedy would be to add them to the essential drugs list so that the cost would be largely reimbursable, but this is not currently under consideration by the Chinese government^34^.

Importantly, the relationship between e-cigarette use and quitting attempts is substantially impacted by nicotine dependence level. Although previous work argues that e-cigarettes may be valuable as a smoking cessation approach^35^, the current findings only support this to some extent. In particular, we did not identify an association between e-cigarette use and positive quitting attempts in moderate- and high-dependence smokers. On the other hand, among those with very low nicotine dependence, we observed an association between e-cigarette use and more positive quitting attempts. However, since the safety and long-term outcomes of e-cigarettes are still uncertain and because we used a cross-sectional design, caution should be used when interpreting these results.

Our study has several limitations. First, we used only cross-sectional data for this estimation. As a result, we cannot infer a causal relationship. Second, all the information we collected was based on self-report without verification, and the respondents’ interpretations of some questions might vary. Third, these analyses are limited by the available variables, especially regarding e-cigarette use. In particular, we did not separate lifetime use and recent users. This may have weakened the effects. Fourth, we may also have unmeasured confounders that contributed to quitting intention, such as chronic disease. Fifth, we assessed quitting behaviour retrospectively, but all other variables currently, which may bias our results. However, because all the information was collected at random, we believe the overall findings are meaningful.

Despite these limitations, our findings have some policy and theoretical implications. First, this study extends existing research in critical ways, primarily in relation to how nicotine dependence may alter the relationship between quitting intention and behaviour. This suggests that enhancing Chinese smokers’ quitting intentions may not lead to quitting attempts for all smokers. Therefore, it is important to make some smoking cessation services available to high-dependence smokers. A suitable plan for quitting should be developed depending on an individual’s level of nicotine dependence. Second, the current findings show a complex relationship between e-cigarette use and quitting attempts that may warrant different research for different types of users. In particular, e-cigarette use may have potential with respect to promoting quitting among low-dependence smokers, but it may be less appropriate to use e-cigarettes as a kind of cessation medicine among highly dependent smokers. Future research using experimental and longitudinal designs is needed to evaluate these preliminary findings.

## Conclusion

The present study revealed that smokers with various levels of nicotine dependence have different psychological determinants of quitting intention. Quitting intention was more significantly associated with quitting behaviour for the low nicotine dependence group than for the other groups. More convincing research is necessary to determine how e-cigarette use affects quitting behaviour. The results of this empirical analysis not only contribute to identifying the determinants of psychological predictors of quitting intention but also provide further evidence about how nicotine dependence affects quitting behaviour among residents in a developing country, thereby adding to earlier research on this topic.

## Supplementary Information

Supplementary Information.

## Data Availability

The data of the studies is accessible via Peking University, School of Public Health.
